# Supplementary material for: Common host variation drives malaria parasite fitness in healthy human red cells
Source: eLife. 2021 Sep 23;10:e69808. doi: 10.7554/eLife.69808 (PMC8497061; doi:10.7554/eLife.69808)
Supplement: Transparent reporting form [file elife-69808-transrepform1.docx]

***eLife’s* transparent reporting form**

We encourage authors to provide detailed information *within their submission* to facilitate the interpretation and replication of experiments. Authors can upload supporting documentation to indicate the use of appropriate reporting guidelines for health-related research (see [EQUATOR Network](http://www.equator-network.org/%20)), life science research (see the [BioSharing Information Resource](https://biosharing.org/)), or the [ARRIVE guidelines](http://www.plosbiology.org/article/info:doi/10.1371/journal.pbio.1000412) for reporting work involving animal research. Where applicable, authors should refer to any relevant reporting standards documents in this form.

If you have any questions, please consult our Journal Policies and/or contact us: [editorial@elifesciences.org](mailto:editorial@elifesciences.org).

**Sample-size estimation**

- You should state whether an appropriate sample size was computed when the study was being designed
- You should state the statistical method of sample size computation and any required assumptions
- If no explicit power analysis was used, you should describe how you decided what sample (replicate) size (number) to use

Please outline where this information can be found within the submission (e.g., sections or figure legends), or explain why this information doesn’t apply to your submission:

In the absence of prior knowledge of the variance in parasite fitness among RBCs from different donors, a formal power analysis was of limited utility. Instead, as stated in the Methods, the sample size was designed to ensure we would capture multiple individuals carrying alleles of moderate frequency (5% or greater).

**Replicates**

- You should report how often each experiment was performed
- You should include a definition of biological versus technical replication
- The data obtained should be provided and sufficient information should be provided to indicate the number of independent biological and/or technical replicates
- If you encountered any outliers, you should describe how these were handled
- Criteria for exclusion/inclusion of data should be clearly stated
- High-throughput sequence data should be uploaded before submission, with a private link for reviewers provided (these are available from both GEO and ArrayExpress)

Please outline where this information can be found within the submission (e.g., sections or figure legends), or explain why this information doesn’t apply to your submission:

The Methods state that for the majority of samples, each experiment was performed once on each sample with three technical replicates and two different strains of *P. falciparum*. Blood samples from eleven non-control donors were drawn a second time and experiments were repeated a second time; these are considered biological replicates. For the parasite assays, three technical replicates were performed; if one replicate was an outlier, it was excluded. For the osmotic fragility tests, two technical replicates were performed; individual outlier points were removed based on the consensus curve. No other data were excluded.

**Statistical reporting**

- Statistical analysis methods should be described and justified
- Raw data should be presented in figures whenever informative to do so (typically when N per group is less than 10)
- For each experiment, you should identify the statistical tests used, exact values of N, definitions of center, methods of multiple test correction, and dispersion and precision measures (e.g., mean, median, SD, SEM, confidence intervals); and, for the major substantive results, a measure of effect size (e.g., Pearson's r, Cohen's d)
- Report exact p-values wherever possible alongside the summary statistics and 95% confidence intervals. These should be reported for all key questions and not only when the p-value is less than 0.05.

Please outline where this information can be found within the submission (e.g., sections or figure legends), or explain why this information doesn’t apply to your submission:

Statistical analysis methods are described in the Methods, as well as in the legends of Figures 2-6. Exact values of N, which are identical for all experiments, are also reported in the first section of the Results.

Data points and curves from individual samples are presented in all six main figures. The data in Figures 2-6 have been normalized, as described in the Methods. Raw data and normalization scripts are available at <https://github.com/emily-ebel/RBC>.

Exact p-values and measures of effect size are reported for every test throughout the manuscript. They are occasionally summarized across multiple tests in the written text (e.g. ‘both p<0.02’), but exact values are available in Source Data and Figure Supplement files.

(For large datasets, or papers with a very large number of statistical tests, you may upload a single table file with tests, Ns, etc., with reference to sections in the manuscript.)

**Group allocation**

- Indicate how samples were allocated into experimental groups (in the case of clinical studies, please specify allocation to treatment method); if randomization was used, please also state if restricted randomization was applied
- Indicate if masking was used during group allocation, data collection and/or data analysis

Please outline where this information can be found within the submission (e.g., sections or figure legends), or explain why this information doesn’t apply to your submission:

Samples were allocated into groups based on their genotypes at known RBC disease loci, as described in the first section of the Results.

**Additional data files (“source data”)**

- We encourage you to upload relevant additional data files, such as numerical data that are represented as a graph in a figure, or as a summary table
- Where provided, these should be in the most useful format, and they can be uploaded as “Source data” files linked to a main figure or table
- Include model definition files including the full list of parameters used
- Include code used for data analysis (e.g., R, MatLab)
- Avoid stating that data files are “available upon request”

Please indicate the figures or tables for which source data files have been provided:

Source data files are provided for the data summarized by tables in Figures 4 and 5. All other raw data and normalization scripts are available at <https://github.com/emily-ebel/RBC>.
